# Triparental care in the collared flycatcher (*Ficedula albicollis*): Cooperation of two females with a cuckolded male in rearing a brood

**DOI:** 10.1002/ece3.7923

**Published:** 2021-07-28

**Authors:** Miklós Laczi, Renáta Kopena, Fanni Sarkadi, Dóra Kötél, János Török, Balázs Rosivall, Gergely Hegyi

**Affiliations:** ^1^ Behavioural Ecology Group Department of Systematic Zoology and Ecology Institute of Biology ELTE Eötvös Loránd University Budapest Hungary; ^2^ The Barn Owl Foundation Orosztony Hungary; ^3^ Centre for Ecological Research Institute of Ecology and Botany Vácrátót Hungary; ^4^ Ecology Research Group of the Hungarian Academy of Sciences Budapest Hungary

**Keywords:** collared flycatcher, cooperative care, microsatellite genotyping, parental care

## Abstract

Certain predominant forms of mating and parental care systems are assumed in several model species among birds, but the opportunistic and apparently infrequent variations of “family structures” may often remain hidden due to methodological limitations with regard to genetic or behavioral observations. One of the intensively studied model species, the collared flycatcher (*Ficedula albicollis*), is usually characterized by social monogamy with polyterritorial, facultative social polygyny, and frequent extrapair mating and extrapair paternity. During a brood‐size manipulation experiment, we observed two females and a male delivering food at an enlarged brood. A combination of breeding phenology data (egg laying and hatching date), behavioral data (feeding rates) from video recordings at 10 days of nestling age, and microsatellite genotyping for maternity and paternity suggests a situation of an unrelated female helping a pair in chick rearing. Such observations highlight the relevance of using traditional techniques and genetic analyses together to assess the parental roles within a population, which becomes more important where individuals may dynamically switch from their main and presupposed roles according to the actual environmental conditions.

## Graphical abstract

The general mating and parental care systems are deeply studied in many model species, but we have only little information on the rare alternative tactics occurring under unforeseen, stressful environmental conditions. In this paper, we took a report on a case of cooperative nestling rearing with alloparental care in the socially monogamous collared flycatcher. Using together breeding phenology, behavioral, and genetic data, we provide a possible explanation for the observed phenomenon.

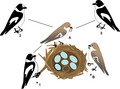

## 1 INTRODUCTION

Passerines are predominantly characterized by social monogamy (Lack, 1968) with posthatching biparental care (Cockburn, 2006), but there are no strict rules of thumb for living according to a certain kind of mating and parental care system (Bennett & Owens, 2002). Hence, there are species where the above systems vary within populations depending on local environmental or social conditions, as in the evergreen case of dunnocks (*Prunella modularis*, Davies & Lundberg, 1984) or in penduline tits (*Remiz pendulinus*, Persson & Öhrström, 1989). In spite of this plasticity, cooperative breeding strategies are apparently quite rare among passerines (Brown, 2014; Ligon, 1999). In these cases, a single brood is cared for by at least two individuals belonging to the same sex (Vehrencamp, 2000). Opportunistic cooperative polygyny or communal laying has been observed in a few species, for example, in pied flycatchers (*Ficedula hypoleuca*, Lifjeld et al., 1992), common starlings (*Sturnus vulgaris*, Eens & Pinxten, 1993), black‐browed reed warblers (*Acrocephalus bistrigiceps*, Hamao & Ueda, 1998), whereas a helper‐at‐the‐nest system is conventional in superb fairywrens (*Malurus cyaneus*, Dunn et al., 1995) and Seychelles warblers (*Acrocephalus sechellensis*, Richardson et al., 2002). In some of these systems, there may be individuals in a “family unit” that help raise the offspring of others, performing alloparental care.

Breeding and parental roles in one of the most investigated model species in behavioral ecology, the collared flycatcher (*Ficedula albicollis*), have seemed to be only moderately plastic. We have been studying a population since the early 1980s (see below for details). At our study site, these birds arrive from spring migration in late April. During a breeding season, females lay one clutch with 5–8 eggs incubated exclusively by them, but nestlings are fed by females and males together. In our study population, only 5.7% of males become socially polygynous during their lifetime (Herényi et al., 2014), based on capture data. Due to female aggression (Hegyi et al., 2007), polyterritoriality typically occurs in polygynous males with a territory distance of 15–300 m. In contrast to the very low rate of social polygyny, experimental mating restriction in combination with sperm counting on the perivitelline layer (Michl et al., 2002) and microsatellite‐based paternity analyses (Rosivall et al., 2009) have revealed a high rate of genetic promiscuity due to extrapair (EP) copulations. Under natural conditions, 56% of the broods contained offspring from EP males, and approx. 21% of the nestlings were sired by EP father (Rosivall et al., 2009). Studies of other populations of this species have clearly shown the absence of intraspecific brood parasitism (Krist et al., 2005; Sheldon & Ellergren, 1996). Löhrl (1957) described that nest adoption by a male could occur at nests which the father had deserted, and he took an anecdotal note that once he observed a nest with one female and two males. In addition to the above patterns, we now provide detailed field and genetic data on the occurrence of one kind of cooperative breeding with three carers (two females and one male) and alloparental care in this species.

## 2 MATERIALS AND METHODS

We collected data from a collared flycatcher population breeding in artificial nest box plots located in the Pilis–Visegrádi Mountains (Duna–Ipoly National Park, Hungary, 47°43′N, 19°01′E), in 2015 and 2016. The study site is covered by deciduous woodland dominated by oaks and consisted of ca. 750 artificial nest boxes (*n* = 747 in 2015, *n* = 707 in 2016), which are used principally by collared flycatchers, great tits, and blue tits (pooling the 2 years, there were 534 collared flycatcher nesting events if including failed ones too). Nest box plots were checked at every 5 days in order to determine laying date, clutch size, and hatching date. During the breeding seasons of 2015 and 2016, in a brood‐size manipulation experiment, we created trios (*n* = 10 in 2015, *n* = 9 in 2016) of certain nests and partially cross‐fostered nestlings between two nests within trios (transferring 2 nestlings from nest A to nest B, and 4 nestlings from B to A; C was nonmanipulated), when the older nestlings of a nest were 2 days old. In our population, clutches generally contain asynchronously hatched nestlings: Usually, at least half of the nestlings hatch on the first day, and the others hatch on the next day. Rarely, 2‐day asynchrony may occur between the oldest and the youngest nestlings, but these clutches could not be included into our brood‐size manipulation experiment, according to our protocol.

We conducted video recordings to collect data on nestling feeding rates (number of visits with food delivery per hour) at the nests of the trios when the older chicks in each nest were 10 days old (*n* = 29 in 2015, *n* = 26 in 2016). As nestlings were of the same age within a trio, video recordings of nests of a trio were conducted on the same day and hour. We performed the video recordings between 0900 and 1830, mostly during the first half of the day. Earlier it had been described for this study population that no difference exists between morning and afternoon feeding rates of parents (Kiss et al., 2013). We avoided recording in midday hours (1200–1400). Recording length was a few minutes more than 1 hr, and we analyzed 60 min of the footages, without the first few 10–15 min. For detailed methods and results, see Laczi et al., 2017. Additionally, for another experiment, video recordings were conducted (with 2‐hr footages) at other nests too with 10‐day‐‐old offspring (*n* = 17 in 2015, *n* = 19 in 2016, see details in Szász et al., 2019).

Just after recording, we set up a spring trap at each nest box. According to our general protocol, we finish trapping after we catch a female and a male bird (supposing that they are the only parents) or after a maximum of 1.5 hr. We ringed the adult birds if they were not ringed formerly. Blood samples (ca. 10–15 µl) were collected from the wing vein of nestlings and the captured parents for the purpose of paternity analysis. Blood samples were stored in absolute ethanol and kept at −20℃ until the analyses. DNA was extracted using an ammonium‐acetate method (Nicholls et al., 2000). We assessed maternity and paternity by using ten polymorphic microsatellite loci (FhU2, FhU4, Fhy405, Fhy407, Fhy428, Fhy429, Fhy431, Fhy452, Cuµ4, Pdoµ5 (Ellegren, 1992; Primmer et al., 1996; Griffith et al., 1999; Gibbs et al., 1999; Leder et al., 2008)). All PCRs were run using the Type‐it microsatellite PCR kit (QIAGEN) and the following thermal profile: 95℃ for 5 min, 30 cycles of 95℃ for 30 s, 56℃ for 30 s, 72℃ for 30 s, and a final step of 60℃ for 30 min.

PCR products were analyzed on ABI 3130xl Genetic Analyzer (Applied Biosystems, Foster City, CA, USA) with a 50 cm capillary and POP‐7 polymer (Applied Biosystems, Foster City, CA, USA) using the internal size standard GeneScan 600 LIZ (Applied Biosystems, Foster City, CA, USA). Fragment lengths were determined using Peak Scanner Software v.1.0 (Applied Biosystems, Foster City, CA, USA).

Maternity and paternity testing of the nestlings was performed manually by the exclusion method (Jones & Ardren, 2003). Assuming Mendelian inheritance, offspring were classified as not related to their putative parents if they did not match at one or more loci.

## 3 RESULTS

Breeding data indicated that the first egg in the nest box in question was laid on 27 April 2016. On 03 May, we found five eggs, which suggested that the last egg was laid on 01 May as females typically lay one egg on each consecutive day. On 07 May, the nest contained seven eggs. This means that there was a gap of a few days in egg laying, with a restart between 04 and 06 May. On May 20, we found six nestlings: three of them were 1 day old and the others were 2 days old, based on their appearances and body masses. We did not find any further propagule, indicating that one egg or hatchling had vanished. This missing offspring could not be sampled for DNA later.

**FIGURE 1:**
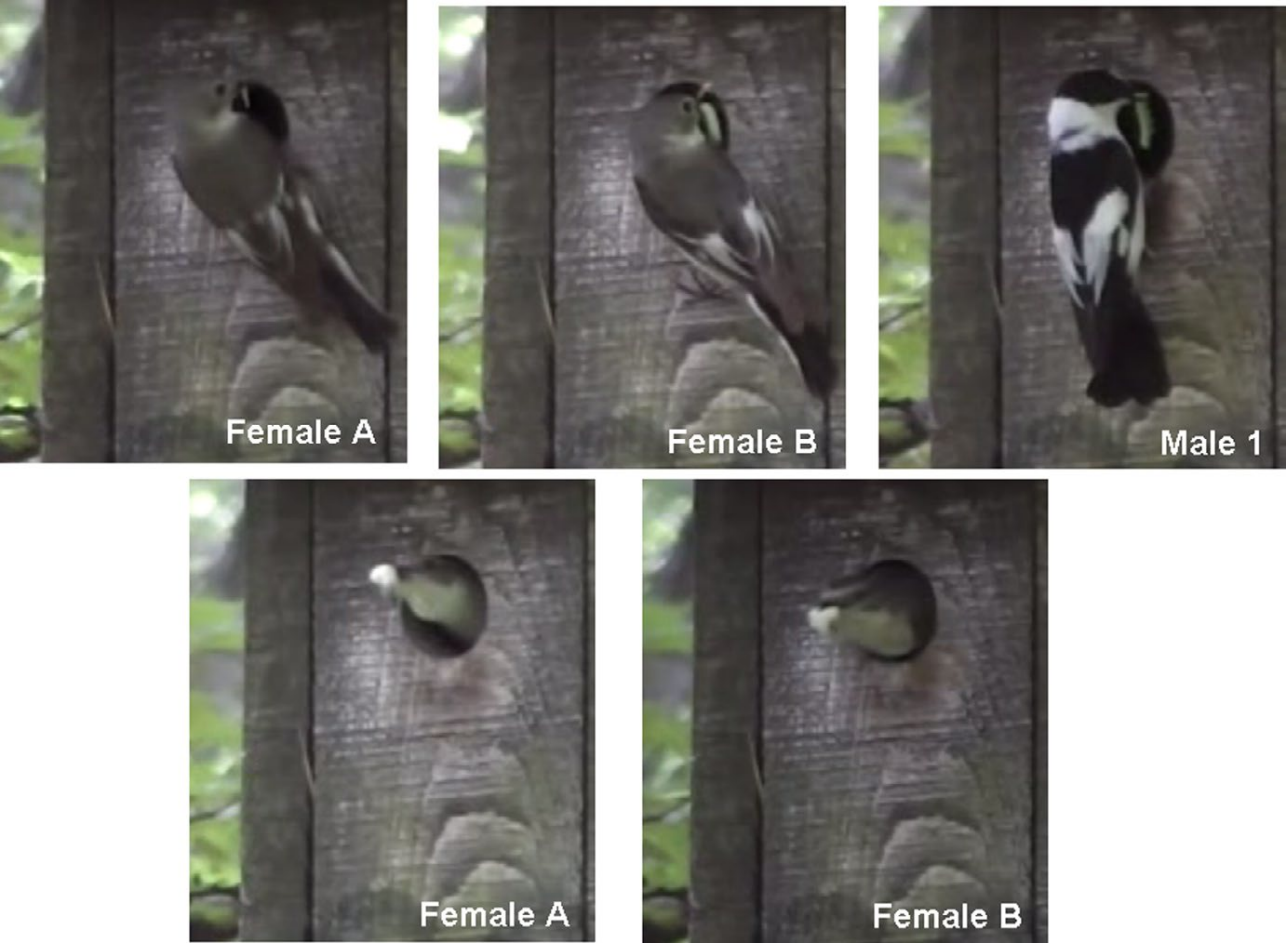
Cooperative parental care at a collared flycatcher nest. Each of the parents delivered food for the nestlings and took out the nestling feces

The nest was subject to a cross‐fostering experiment, and it was enlarged by three nestlings (from six to nine). Processing the video records, we observed that two females, which were distinguished by their different plumage patterns, and one male (Figure 1.) were visiting the nest regularly. During the 1‐hr record we analyzed, we found that female (A) brought prey items 23 times, female (B) 15 times, and the male 28 times. The mean feeding rate of the other males was as follows: 19.31/22.13/32.81 in reduced/control/enlarged broods, for other females, it was 17.50/24.75/34.31, respectively (see Laczi et al., 2017). The two females attending this brood were standing together inside the nest box three times.

**FIGURE 2:**
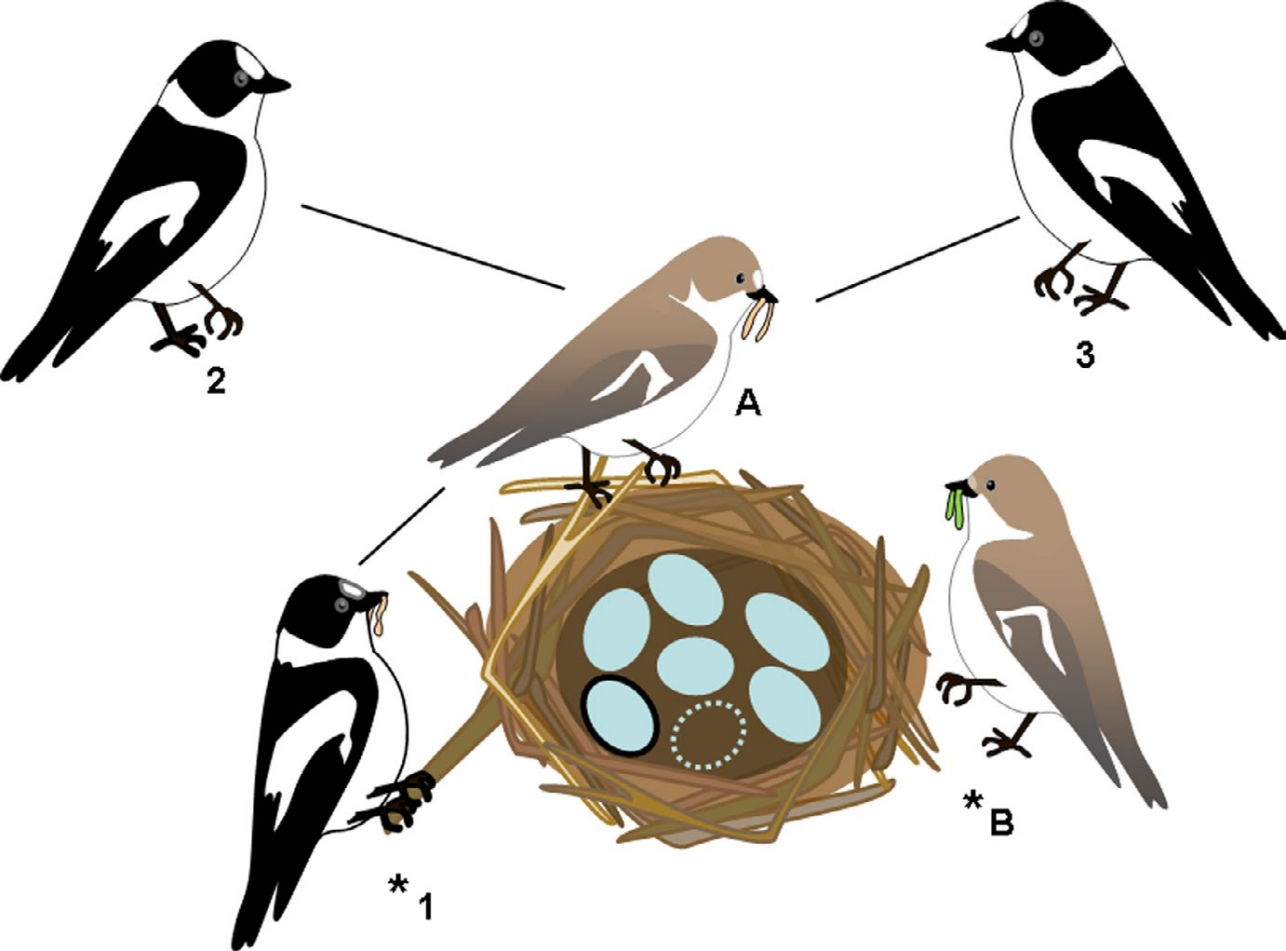
Relationships within an unusual collared flycatcher family with cooperative parental care. Video record revealed that three individuals (females (A) and (B), male (1)) were regularly delivering food and were taking out nestling feces. Microsatellite fingerprinting of the captured adults (marked with asterisks) and nestlings suggested that the captured female (B) was unrelated to the progenies, and probably all offspring shared one genetic mother, female (A). The captured male (1) was genetically related to only one offspring, represented by an egg with thick outline. The other nestlings were sired by at least two other males (2) and (3). Lines connect individuals with genetically shared offspring. Empty ellipse represents an egg that disappeared and was not sampled. For more details, see the text

The genetic analysis of the six offspring that remained and the captured adults suggests that the captured female was related to none of the nestlings. On each locus, we could identify two alleles, either of which was present in every offspring. The most likely scenario is therefore that all nestlings shared the same mother (which we did not catch), even though we cannot completely exclude the existence of multiple mothers. The captured male sired only one progeny, and, assuming one mother, the other nestlings were sired by at least two other males. The captured female and male were unrelated or very distantly related to each other. The alleles of captured female, male, and offspring together with the assumed alleles of the putative mother are indicated in Table 1. Figure 2 shows a graphical representation of this unusual family setup.

**TABLE 1:** Genetic profiles of the captured female, male, the nestlings, and the potential profile of the putative mother at ten microsatellite loci

	FhU2		Fhy431		Fhy428		Cuu4		Fhy407		Fhy452		Fhy405		FhU4		Pdou5		Fhy429	
	a	b	a	b	a	b	a	b	a	b	a	b	a	b	a	b	a	b	a	b
Captured female	149	161	220	232	289	293	133	145	215	227	318	352	155	159	181	189	223	235	309	352
Captured male	159	161	224	228	293	301	145	157	211	219	290	368	128	175	177	177	229	231	319	432
Nestling 1	139	**159**	221	**224**	285	**293**	141	**145**	211	**211**	314	**368**	147	**175**	**177**	189	**229**	**231**	307	**319**
Nestling 2	159	167	221	225	277	331	135	137	211	243	304	314	147	155	177	197	231	233	315	321
Nestling 3	139	149	221	224	285	301	131	137	211	219	290	314	159	162	195	197	228	231	305	307
Nestling 4	139	149	216	228	277	289	131	141	211	219	290	314	147	151	177	197	228	231	315	321
Nestling 5	139	167	224	228	277	301	131	137	211	219	290	314	159	162	197	197	228	231	305	321
Nestling 6	149	167	221	236	277	285	137	141	211	235	314	332	155	159	181	197	231	235	321	327
Putative mother	139	167	221	228	277	285	137	141	211	*	314	*	147	159	189	197	231	*	321	307

Note

The alleles were identified by capillary gel electrophoresis. Only one nestling was sired by the captured male (nestling 1; alleles that could be transmitted by the male are indicated in bold). At 3 loci, only one allele was present in every offspring; therefore, we could not identify the other alleles of these loci (marked with asterisks) for the putative mother.

## 4 DISCUSSION

In our case study, we detected cooperative parental care with two females and one male in the collared flycatcher, a species previously described as showing social monogamy (or polyterritorial polygyny) and biparental care. Neither of the two females nor the male that appeared on the video record seemed to be a prospector, because all of them showed active parental behavior in terms of providing nourishment and taking out nestling feces several times, and none of these are attributes of prospective behavior in this species (Doligez et al., 2004). Feeding rate of the male was similar to the mean feeding rate of the other males that raised enlarged broods. In contrast, neither of the females showed the expected elevated feeding intensity. However, the two females together fed at a total rate (38) similar to the mean feeding rate of socially monogamous females of enlarged broods. It is clear that in spite of the presence of two females' assistance, the male's feeding intensity did not reduce, as well as from this triparental care the nestlings did not gain extra advantages at least with regard to their mean body mass at 12 days of age, close to fledging (12.4 g; in other enlarged broods: 12.7 g, Laczi et al., 2017).

The original nest contained eggs that had been laid in two separate turns. Based on this and the behavioral observations, one would conceive that the parenting mothers shared the genetic maternity too. If we take a look at the microsatellite patterns, we can draw quite a different conclusion, namely that there was probably only one genetic mother. This is also supported by the highly synchronous hatching (at most 1‐day difference between the older and the younger nestlings) which suggested that the incubation did not start until after the laying of the fifth egg. The occurrence of laying gaps may be related to abiotic environmental stress conditions (Eeva & Lehikoinen, 2010; Nilsson & Svensson, 1993) or to social interactions (Low, 2008). Here, disturbances by predators may have led to the interruption in egg laying as there were nest predation events around this nest box during that breeding season (caused most probably by European pine marten (*Martes martes*) or beech marten (*M. foina*) based on the clues), aborting nesting in multiple nest boxes that had already contained eggs or nestlings.

The most likely cause of the appearance of the second, that is, the captured female (B), is also predation. Perhaps the inner drive of the female was so strong that after the depredation of her own nest, she continued feeding in a nearby nest box where hungry nestlings begged for food. In this case, the observed behavior was simply a sequel of reproductive error (Riedman, 1982) and did not provide any benefits. Alternatively, it might be beneficial in terms of using resources, or group membership may have enhanced antipredator defense (Emlen et al., 1991; Riedman, 1982). In any case, the lack of aggression between the two females (i.e., neither female chased away the other) suggests that the second female had already been parenting for a longer time and did not begin only on the day of the video recording.

With regard to the male, it has been shown in another collared flycatcher population that socially monogamous males adjust their provisioning rates according to their assumed share of paternity (Sheldon & Ellegren, 1998). We cannot completely exclude that the usually representative 1‐hr sampling of parental care may have accidentally fallen on an unusual period at this particular nest. However, if the recorded care pattern is representative, we would speculate based on the feeding rate of the captured male that it was the genetic father of the majority of the original nestlings. By contrast, DNA analyses have shown us that most of the offspring were not sired by the captured male. In socially monogamous bird species, males that engage in extrapair copulations usually do not care for their offspring sired in other than their social mate's nest (Birkhead & Møller, 1992). In addition, when we estimated the height of the forehead patch of the male on the video record (using box entrance diameter as reference by Scanning Probe Image Processor, Image Metrology, Inc.), it was almost identical to the measured forehead patch size of the captured male (9.0 and 9.1 mm, respectively). Thus, we supposed that the captured male was identical to the one observed on the video record. If so, based on the genetic data, he must have been cuckolded by other males. In a socially monogamous system with extrapairs, it is not a curiosity if a male raises a nest with only one or even no own progeny (Gibbs et al., 1990; Kempenaers et al., 2001).

In conclusion, this brood was apparently cared for by a female that was related to none of the nestlings, the actual mother of the nestlings who had obtained extrapair fertilizations from at least two foreign males, and her heavily cuckolded social mate. Our finding is in accordance with the phenomenon that certain adverse environmental factors, such as elevated intensity of local predation, may dynamically rearrange the general mating and parental care scenario among a subset of individuals within a population. For example, in the socially monogamous blue tit (*Cyanistes caeruleus*), unbalanced sex ratio of reproductive birds, for example, the death of a neighboring parental male, may give rise to replacement polygyny (Kempenaers, 1994). In the same species, failed breeding could cause the type of nest adoption (Santema & Kempenaers, 2021) which has also been described in the collared flycatcher (Löhrl, 1957) (see Introduction). Mating and parental care relationships can be flexibly fine‐tuned in accordance with availability of either sex not only in birds, but also in mammals, as described, for example, in the wild boar (*Sus scrofa*), where hunting on males reduced the degree of polygyny (Poteaux et al., 2009).

Our observations convey a warning message for investigations of reproductive behavioral patterns. After capturing a female and a male of a species that is expected to be socially monogamous and biparental, it may not be justified to automatically suppose that they are the mates of each other, and they are the parents or the only parents. As another study on the pied flycatcher suggested, applying various methods together (i.e., genetic and behavioral) may be required to dig out the actual truth in some uncertain cases (Grinkov et al., 2018), thereby avoiding the misinterpretation caused by using one method only. Hence, a holistic approach can be highly beneficial when estimating actual mating preferences and realized reproductive success.

## CONFLICT OF INTEREST

The authors declare no competing interest.

## AUTHOR CONTRIBUTIONS

**Miklós Laczi:** Conceptualization (lead); data curation (lead); investigation (equal); visualization (lead); writing–original draft (lead); writing–review and editing (lead). **Renáta Kopena:** Conceptualization (equal); formal analysis (equal); investigation (equal); visualization (equal); writing–review and editing (equal). **Fanni Sarkadi:** Conceptualization (equal); formal analysis (equal); investigation (equal); visualization (equal); writing–review and editing (equal). **Dóra Kötél:** Data curation (equal); investigation (equal); visualization (equal); writing–review and editing (equal). **János Török:** Conceptualization (equal); supervision (equal); visualization (equal); writing–review and editing (equal). **Balázs Rosivall:** Conceptualization (equal); formal analysis (equal); funding acquisition (equal); resources (equal); visualization (equal); writing–review and editing (equal). **Gergely Hegyi:** Conceptualization (equal); funding acquisition (lead); investigation (equal); project administration (equal); resources (equal); supervision (equal); visualization (equal); writing–review and editing (equal).

## Data Availability

All data are included in the publication.

## References

[ece37923-bib-0001] Bennett, P. M., & Owens, I. P. (2002). Evolutionary ecology of birds: Life histories, mating systems and extinction. (Oxford Series in Ecology and Evolution) Oxford University Press.

[ece37923-bib-0002] Birkhead, T. M., & Møller, A. P. (1992). Sperm competition in birds: Evolutionary causes and consequences. Academic Press.

[ece37923-bib-0003] Brown, J. L. (2014). Helping communal breeding in birds: Ecology and evolution. Princeton University Press.

[ece37923-bib-0004] Cockburn, A. (2006). Prevalence of different modes of parental care in birds. Proceedings of the Royal Society B: Biological Sciences, 273, 1375–1383. 10.1098/rspb.2005.3458 PMC1560291 16777726

[ece37923-bib-0005] Davies, N. B., & Lundberg, A. (1984). Food distribution and a variable mating system in the dunnock, *Prunella modularis* . The Journal of Animal Ecology, 53, 895–912. 10.2307/4666

[ece37923-bib-0006] Doligez, B., Pärt, T., & Danchin, E. (2004). Prospecting in the collared flycatcher: Gathering public information for future breeding habitat selection? Animal Behaviour, 67, 457–466. 10.1016/j.anbehav.2003.03.010

[ece37923-bib-0007] Dunn, P. O., Cockburn, A., & Mulder, R. A. (1995). Fairy‐wren helpers often care for young to which they are unrelated. Proceedings of the Royal Society of London. Series B: Biological Sciences, 259, 339–343.

[ece37923-bib-0008] Eens, M., & Pinxten, R. (1993). Female starlings sharing the same nestbox. Ringing, & Migration, 14, 135–136. 10.1080/03078698.1993.9674057

[ece37923-bib-0009] Eeva, T., & Lehikoinen, E. (2010). Polluted environment and cold weather induce laying gaps in great tit and pied flycatcher. Oecologia, 162, 533–539. 10.1007/s00442-009-1468-9 19784674

[ece37923-bib-0010] Ellegren, H. (1992). Polymerase‐chain‐reaction (PCR) analysis of microsatellites: A new approach to studies of genetic relationship in birds. The Auk, 109, 886–895.

[ece37923-bib-0011] Emlen, S. T., Reeve, H. K., Sherman, P. W., Wrege, P. H., Ratnieks, F. L., & Shellman‐Reeve, J. (1991). Adaptive versus nonadaptive explanations of behavior: The case of alloparental helping. The American Naturalist, 138, 259–270. 10.1086/285216

[ece37923-bib-0012] Gibbs, H. L., Tabak, L. M., & Hobson, K. (1999). Characterization of microsatellite DNA loci for a neotropical migrant songbird, the Swainson’s thrush (*Catharus ustulatus*). Molecular Ecology, 8, 1551–1552. 10.1046/j.1365-294x.1999.00673.x 10564463

[ece37923-bib-0013] Gibbs, H. L., Weatherhead, P. J., Boag, P. T., White, B. N., Tabak, L. M., & Hoysak, D. J. (1990). Realized reproductive success of polygynous red‐winged blackbirds revealed by DNA markers. Science, 250, 1394–1397. 10.1126/science.250.4986.1394 17754986

[ece37923-bib-0014] Griffith, S. C., Stewart, I. R. K., Dawson, D. A., Owens, I. P. F., & Burke, T. (1999). Contrasting levels of extra‐pair paternity in mainland and island populations of the house sparrow (*Passer domesticus*): Is there an ‘island effect’? The Biological Journal of the Linnean Society, 68, 303–316. 10.1111/j.1095-8312.1999.tb01171.x

[ece37923-bib-0015] Grinkov, V. G., Bauer, A., Gashkov, S. I., Sternberg, H., & Wink, M. (2018). Diversity of social‐genetic relationships in the socially monogamous pied flycatcher (*Ficedula hypoleuca*) breeding in Western Siberia. PeerJ, 6, e6059. 30564520 10.7717/peerj.6059 PMC6286800

[ece37923-bib-0016] Hamao, S., & Ueda, K. (1998). Nest sharing by polygynously mated females in the Black‐browed Reed Warbler *Acrocephalus bistrigiceps* . Ibis, 140, 176–178. 10.1111/j.1474-919X.1998.tb04558.x

[ece37923-bib-0017] Hegyi, G., Rosivall, B., Szöllősi, E., Hargitai, R., Eens, M., & Török, J. (2007). A role for female ornamentation in the facultatively polygynous mating system of collared flycatchers. Behaviroal Ecology, 18, 1116–1122. 10.1093/beheco/arm085

[ece37923-bib-0018] Herényi, M., Garamszegi, L. Z., Hargitai, R., Hegyi, G., Rosivall, B., Szöllősi, E., & Török, J. (2014). Laying date and polygyny as determinants of annual reproductive success in male collared flycatchers (*Ficedula albicollis*): A long‐term study. Naturwissenschaften, 101, 305–312. 10.1007/s00114-014-1157-3 24563121

[ece37923-bib-0019] Jones, A. G., & Ardren, W. R. (2003). Methods of parentage analysis in natural populations. Molecular Ecology, 12, 2511–2523. 10.1046/j.1365-294X.2003.01928.x 12969458

[ece37923-bib-0020] Kempenaers, B. (1994). Polygyny in the blue tit: Unbalanced sex ratio and female aggression restrict mate choice. Animal Behaviour, 47, 943–957. 10.1006/anbe.1994.1126

[ece37923-bib-0021] Kempenaers, B., Everding, S., Bishop, C., Boag, P., & Robertson, R. J. (2001). Extra‐pair paternity and the reproductive role of male floaters in the tree swallow (*Tachycineta bicolor*). Behavioral Ecology and Sociobiology, 49, 251–259. 10.1007/s002650000305

[ece37923-bib-0022] Kiss, D., Hegyi, G., Török, J., & Rosivall, B. (2013). The relationship between maternal ornamentation and feeding rate is explained by intrinsic nestling quality. Behavioral Ecology and Sociobiology, 67, 185–192. 10.1007/s00265-012-1437-x

[ece37923-bib-0023] Krist, M., Nádvorník, P., Uvírová, L., & Bureš, S. (2005). Paternity covaries with laying and hatching order in the collared flycatcher *Ficedula albicollis* . Behavioral Ecology and Sociobiology, 59, 6–11. 10.1007/s00265-005-0002-2

[ece37923-bib-0024] Lack, D. (1968). Ecological adaptations for breeding in birds. Methuen.

[ece37923-bib-0025] Laczi, M., Kötél, D., Török, J., & Hegyi, G. (2017). Mutual plumage ornamentation and biparental care: Consequences for success in different environments. Behavioral Ecology, 28, 1359–1368. 10.1093/beheco/arx099

[ece37923-bib-0026] Leder, E. H., Karaiskou, N., & Primmer, C. R. (2008). Seventy new microsatellites for the pied flycatcher, *Ficedula hypoleuca* and amplification in other passerine birds. Molecular Ecology Resources, 8, 874–880. 21585917 10.1111/j.1755-0998.2008.02096.x

[ece37923-bib-0027] Lifjeld, J. T., Breiehagen, T., & Lampe, H. M. (1992). Pied flycatchers failed to use nestling size as a cue to favour own genetic offspring in a communally raised brood. Ornis Scandinavica, 23, 199–201. 10.2307/3676450

[ece37923-bib-0028] Ligon, J. D. (1999). The evolution of avian breeding systems (Oxford Ornithology Series). Oxford University Press.

[ece37923-bib-0029] Löhrl, H. (1957). Populationsökologische Untersuchungen beim Halsbandschnäpper (*Ficedula albicollis*). Bonner Zoologische Beiträge, 8, 130–177.

[ece37923-bib-0030] Low, M. (2008). Laying gaps in the New Zealand Stitchbird are correlated with female harassment by extra‐pair males. Emu‐Austral Ornithology, 108, 28–34. 10.1071/MU07037

[ece37923-bib-0031] Michl, G., Török, J., Griffith, S. C., & Sheldon, B. C. (2002). Experimental analysis of sperm competition mechanisms in a wild bird population. Proceedings of the National Academy of Sciences, 99, 5466–5470. 10.1073/pnas.082036699 PMC122792 11943862

[ece37923-bib-0032] Nicholls, J. A., Double, M. C., Rowell, D. M., & Magrath, R. D. (2000). The evolution of cooperative and pair breeding in thornbills Acanthiza (*Pardalotidae*). Journal of Avian Biology, 31, 165–176.

[ece37923-bib-0033] Nilsson, J. Å., & Svensson, E. (1993). The frequency and timing of laying gaps. Ornis Scandinavica, 122–126. 10.2307/3676361

[ece37923-bib-0034] Persson, O., & Öhrström, P. (1989). A new avian mating system: Ambisexual polygamy in the penduline tit *Remiz pendulinus* . Ornis Scandinavica, 20, 105–111. 10.2307/3676876

[ece37923-bib-0035] Poteaux, C., Baubet, E., Kaminski, G., Brandt, S., Dobson, F. S., & Baudoin, C. (2009). Socio‐genetic structure and mating system of a wild boar population. Journal of Zoology, 278, 116–125. 10.1111/j.1469-7998.2009.00553.x

[ece37923-bib-0036] Primmer, C. R., Møller, A. P., & Ellegren, H. (1996). A wide‐range survey of crossspecies microsatellite amplification in birds. Molecular Ecology, 5, 365–378. 10.1111/j.1365-294X.1996.tb00327.x 8688957

[ece37923-bib-0037] Richardson, D. S., Burke, T., & Komdeur, J. (2002). Direct benefits and the evolution of female‐biased cooperative breeding in Seychelles warblers. Evolution, 56, 2313–2321. 10.1111/j.0014-3820.2002.tb00154.x 12487360

[ece37923-bib-0038] Riedman, M. L. (1982). The evolution of alloparental care and adoption in mammals and birds. The Quarterly Review of Biology, 57, 405–435. 10.1086/412936

[ece37923-bib-0039] Rosivall, B., Szöllősi, E., Hasselquist, D., & Török, J. (2009). Effects of extrapair paternity and sex on nestling growth and condition in the collared flycatcher, *Ficedula albicollis* . Animal Behaviour, 77, 611–617. 10.1016/j.anbehav.2008.11.009

[ece37923-bib-0040] Santema, P., & Kempenaers, B. (2021). Offspring provisioning by extra‐pair males in blue tits. Journal of Avian Biology, 2021, e02755.

[ece37923-bib-0041] Sheldon, B. C., & Ellegren, H. (1998). Paternal effort related to experimentally manipulated paternity of male collared flycatchers. Proceedings of the Royal Society of London. Series B: Biological Sciences, 265, 1737–1742.

[ece37923-bib-0042] Sheldon, B. C., & Ellergren, H. (1996). Offspring sex and paternity in the collared flycatcher. Proceedings of the Royal Society of London. Series B: Biological Sciences, 263, 1017–1021.

[ece37923-bib-0043] Szász, E., Markó, G., Hegyi, G., Török, J., Garamszegi, L. Z., & Rosivall, B. (2019). Nest‐site defence aggression during courtship does not predict nestling provisioning in male collared flycatchers. Behavioral Ecology and Sociobiology, 73, 62. 10.1007/s00265-019-2672-1

[ece37923-bib-0044] Vehrencamp, S. L. (2000). Evolutionary routes to joint‐female nesting in birds. Behavioral Ecology, 11, 334–344. 10.1093/beheco/11.3.334

